# The Enhanced Photo-Electrochemical Detection of Uric Acid on Au Nanoparticles Modified Glassy Carbon Electrode

**DOI:** 10.1186/s11671-017-2225-3

**Published:** 2017-07-14

**Authors:** Yuting Shi, Jin Wang, Shumin Li, Bo Yan, Hui Xu, Ke Zhang, Yukou Du

**Affiliations:** Soochow University, Suzhou, China

**Keywords:** AuNPs, Visible light, Uric acid

## Abstract

In this work, a sensitive and novel method for determining uric acid (UA) has been developed, in which the glassy carbon electrode (GCE) was modified with electrodeposition Au nanoparticles and used to monitor the concentration of UA with the assistant of visible light illumination. The morphology of the Au nanoparticles deposited on GCE surface were characterized by scanning electron microscope (SEM) and the nanoparticles were found to be well-dispersed spheres with the average diameter approaching 26.1 nm. A series of cyclic voltammetry (CV) and differential pulse voltammetry (DPV) measurements have revealed that the introduction of visible light can greatly enhance both the strength and stability of response current due to the surface plasmon resonance (SPR). Specifically, the DPV showed a linear relationship between peak current and UA concentration in the range of 2.8 to 57.5 μM with the equation of *I*_pa_ (μA) = 0.0121*c*_UA_ (μM) + 0.3122 (*R* ^2^ = 0.9987). Herein, the visible light illuminated Au/GCE possesses a potential to be a sensitive electrochemical sensor in the future.

**Electronic supplementary material:**

The online version of this article (doi:10.1186/s11671-017-2225-3) contains supplementary material, which is available to authorized users.

## Background

Uric acid (UA) is one of the terminal products of purine metabolism and present in biological fluids, such as blood and urine. The abnormalities of uric acid levels suggesting the possibility of several diseases, such as gout, kidney disease, hypertension, and cardiovascular, which are associated with elevated uric acid levels, whereas a reduced UA level arouses diseases like multiple sclerosis, Parkinson’s disease, Alzheimer’s disease, and optic neuritis [1]. Therefore, monitoring UA concentration in human body fluid is clinically valuable and significant for the precaution of aforementioned or other similar diseases.

Since the supervision of uric acid concentration in human body fluid is of great significance, various detecting techniques have been developed according to the previous reports, such as enzymatic assay [2, 3], high-performance liquid chromatography (HPLC) [4], mass spectrometry [5], capillary electrophoresis (CE) [6], chemiluminescence [7], and colorimetry [8, 9]. However, all of these traditional methods may need complicated pretreatments or consume too much time. Because of the unique electrochemical activity, uric acid can be irreversibly oxidized into allantoin in aqueous solution so that numbers of researches have focused on quantitating uric acid via electrochemistry methods and the electrochemical sensors are recognized to be promising alternatives to traditional methods. Researches in this area sprung up in recent years, and many novel sensors have been engineered for the detection of UA, such as palladium nanoparticle-loaded carbon nanofibers modified electrode (Pd/CNF-CPE) [10], carbon ionic liquid electrode(CILE) [11], PtAu hybrid film modified electrode [12], chitosan–graphene modified electrode [13], etc. Non-enzymatic methods are been used for direct electrochemical detection of UA, and the mechanism was demonstrated as bellow [14, 15]:
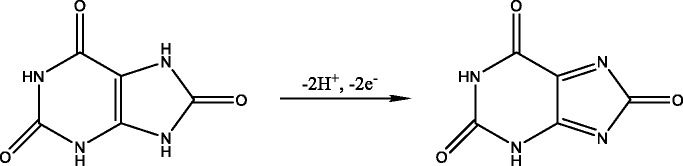


In spite of many endeavors in UA detection, the more sensitive, selective, stable, and facile methods still need to be explored until now.

Due to the superior properties in both chemical and physical aspects, many kinds of nanometals, such as silver (Ag) [16, 17], gold (Au) [18, 19], platinum (Pt) [20–23], copper (Cu) [24, 25], and palladium (Pd) [26, 27] have accelerated numerous researches in the fabrication of electrochemical sensors for medical analysis, environmental monitoring, food quality control, etc. [28]. Especially, gold nanoparticles (AuNPs) show the superiority of conductivity, large surface area, and good biocompatibility [29] and all these unique properties make AuNPs suitable choice for electrochemical sensing. Besides, the unique surface plasmon resonance (SPR) effect of Au for harvesting visible light via the collective coherent oscillation of surface electrons may be beneficial for the great enhancement of detection of UA [30].

Based upon above analyses, we herein demonstrated a facile electrodeposition method to successfully fabricate well-dispersed sphere-like AuNPs on the surface of glassy carbon electrode (GCE). Moreover, a series of electrochemical measurements have revealed that the fascinating Au/GCE exhibited greatly enhanced response current intensity with the assistance of visible light irradiation. In view of these, we believed that this facile visible light assisted method and the spherical Au nanoparticles shed light for the application in the further UA detection and beyond.

## Methods

### Apparatus

The scanning electron microscope (SEM) (Hitachi SU8010, Japan) was used to characterize the morphology of the obtained electrode. All electrochemical studies were performed on a CHI760D electrochemical workstation (Chenhua Instrument Co., Ltd., Shanghai. China) with a conventional three-electrode system where the modified glassy carbon electrode (GCE) acted as the working electrode (surface area 0.07 cm^2^), a platinum wire served as the counter electrode (diameter 0.5 mm), and a saturated calomel electrode (SCE) worked as the reference electrode, respectively. The pH value of solution was determined by a model pHS-2F meter (Instrument, Shanghai, China).

### Reagents

Uric acid (UA) was purchased from Acros Organics (Shanghai, China). Chloroauric acid (HAuCl_4_·H_2_O), disodium hydrogen phosphate (Na_2_HPO_4_), sodium dihydrogen phosphate (NaH_2_PO_4_), potassium ferrocyanide (K_4_[Fe(CN)_6_]), potassium ferricyanide (K_3_[Fe(CN)_6_]), and potassium chloride (KCl) were obtained from Sinopharm Chemicals Reagent Co., Ltd.. Double distilled water was used throughout the experiments. Newly prepared 0.1 M phosphate buffer solution (PBS) of different pH was chosen to be the electrolyte. All the reagents were used as achieved without other purification, and all the experiments were carried out at room temperature.

### Electrode Preparation

In this work, we chose an L-type glassy carbon electrode for the convenience to expose the surface modified with Au nanoparticles to visible light illumination. The GCE (surface area 0.07 cm^2^) was polished carefully with 0.05 μm alumina slurry on a wet polishing cloth followed by ultrasonication in ethanol and water for several minutes to obtain a mirror-like surface. Finally, the electrode was rinsed thoroughly with secondary distilled water and dried in air. Before the follow-up electrochemical process, we polished and rinsed the electrode repeatedly until the cyclic voltammetric curves (CVs) in 2.5 mM Fe(CN)_6_^3/4−^ + 0.1 M KCl solution exhibit two perfect symmetrical peaks and the peak potential separation (Δ*E*_p_) of them was inferior to 100 mV. Au nanoparticles were electrodeposited on the surface of the pre-treated electrodes in 1 mM H_3_PO_4_ solution containing 0.24 mM HAuCl_4_ at a constant potential of −0.2 V. Different mass loading of AuNPs was controlled by changing the electric quantity during the process of deposition.

## Results and Discussion

### Morphology and Characterization of Electrode

**Fig. 1:**
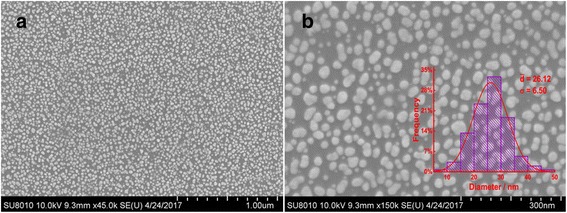
SEM images of Au/GCE at lower (**a**) and higher magnification (**b**). *Inset*: corresponding Au nanoparticles size distribution histogram

The scanning electron microscope (SEM) was used to characterize the morphology of Au nanoparticles on the surface of GCE. The Au nanoparticles were formed at a constant potential of −0.2 V with a certain electric quantity of 3 × 10^−3^ C, and the theoretical density of Au particles on GCE surface was calculated to be 28.9 μg/cm^2^. Figure 1a shows that the GCE surface is densely covered by dot-like gold nanoparticles. Figure 1b presents the surface morphology at a higher magnification and shows that the mellow and spherical AuNPs disperse uniformly on the surface of GCE. The particle size distribution histogram depicted in the inset of Fig. 1b revealed that the well-dispersed AuNPs have a narrow size distribution and the calculated average diameter was approximately 26.1 nm. A detailed observation may also help us find that the as-obtained AuNPs are almost monodispersed, which showed fewer connections with each other. In all, these favorable terms were conductive to greatly increase the active surface area and ultimately lead to the enhancement of detection efficiency.

### Cyclic Voltammetric Behaviors

After modified by Au, CV in 2.5 mM Fe(CN)_6_^3/4−^ + 0.1 M KCl solution was taken to compare with bare GCE (Additional file 1: Figure S1). The CV curve of Au/GCE shows a pair of symmetrical redox peaks, presenting higher response current and smaller peak potential separation (Δ*E*p = 63 mV) than that of bare GCE. From the data above, we can conclude that the electron transfer on Au/GCE was better than that on bare GCE.

**Fig. 2:**
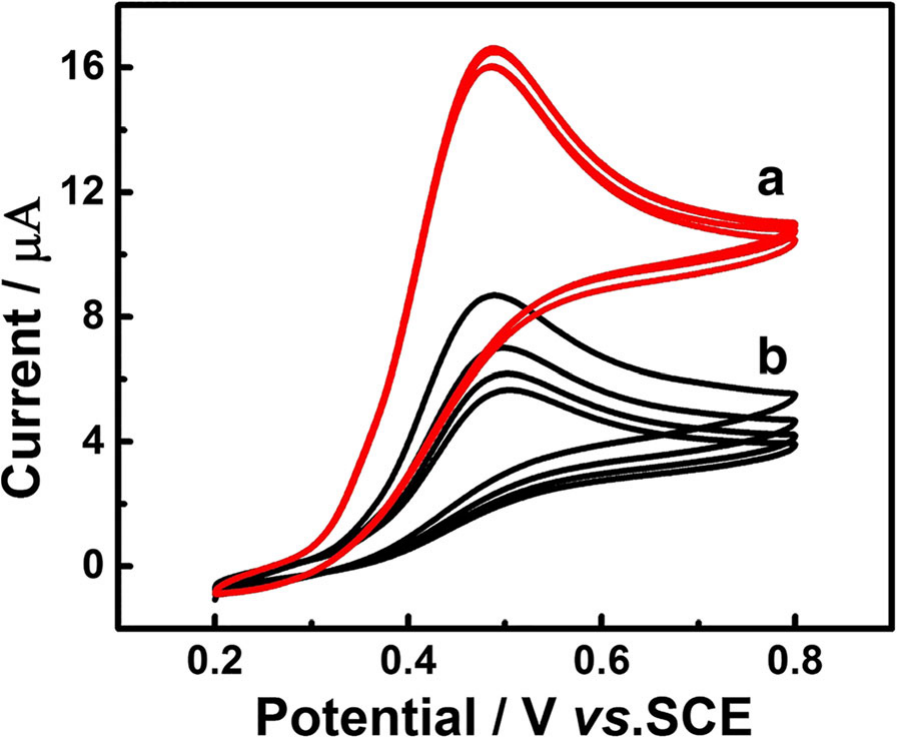
CVs of Au/GCE in 0.1 M PBS (pH 7.0) solution containing 1.0 mM UA with *(a)* and without *(b)* visible light illumination. Scan rate 50 mV s^−1^

Figure 2 shows the CV curves of Au/GCE in 0.1 M PBS (pH 7.0) solution containing 1.0 mM UA with (a) and without (b) visible light illumination for four circles, respectively. We found that when the distance from the xenon lamp to the surface of work electrode is set as 20 cm, the most powerful visible light intensity would be presented. In detail, the anodic peak current for UA was 20 μA under the visible light illumination, while the maximum of the anodic peak current without visible light illumination was only 12 μA. Moreover, with the presence of visible light, the peak current maintained a stable value; nevertheless, the value of the peak current showed a continuous declination without visible light illumination. Besides, the oxidation peak potential of Au/GCE under visible light illumination is approximately 6 mV negative than that without visible light illumination. From above investigations, we can conclude that visible light can improve the photo-electrocatalytic performance of Au/GCE toward electrooxidation of UA.

### Effect of Visible Light Illumination

**Fig. 3:**
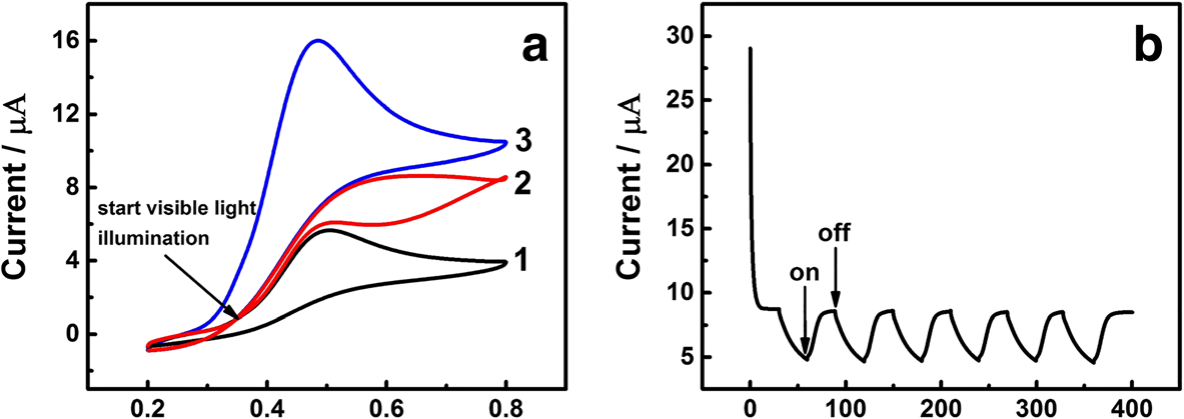
**a** Potential/V vs.SCE. The CV of Au/GCE in 0.1 M PBS (pH 7.0) solution containing 1.0 mM UA without *(1)* and with *(3)* visible light illumination; visible light illumination starts from the spot where the *arrow* is pointing *(2)*. Scan rate 50 mV s^−1^. **b** Time/s. Photocurrent response of Au/GCE under visible light illumination in 0.1 M PBS (pH 7.0) solution containing 1.0 mM UA at 0.48 V. The illumination from a Xe lamp was interrupted every 30 s

The influence of visible light illumination was studied by adding the visible light illumination at a certain time in the process of a successive cyclic voltammetry. As shown in Fig. 3a, the visible light started from the spot where the arrow is pointing. Curve 1 was the last lap of the CVs without visible light illumination. Curve 2 was the transition process, in which the visible light illumination started from the potential of approximately 0.35 V, where the current started to increase theoretically according to Fig. 2. When the electrode is exposed to the visible light illumination, the current presented an ascending tendency, and the CV curve of reverse scan section almost overlaps the curve under the visible light illumination. CV curve under completely illumination of visible light was exhibited as curve 3, as narrating above, the current still maintained steadily. Figure 3b shows photocurrent response of Au/GCE under visible light illumination in 0.1 M PBS (pH 7.0) solution containing 1.0 mM UA at the potential of 0.48 V, at which the current reaches to its maximum in Fig. 2. The photocurrents of the as-prepared electrodes were followed by current–time (*I*–*t*) curves. In order to compare the light and no light conditions for UA detection, we interrupted the Xe lamp illumination periodically. It can be found that while the illumination was interrupted, the photocurrent dropped instantly, and the photocurrent increased quickly when turn on the illumination, and then reached to the equilibrium value in a short time. The perfect periodicity demonstrated in the picture indicated that the response to the visible light illumination is steady and the time required for the response is equal. The surface plasmon resonance (SPR) effect of Au nanoparticles under the visible light illumination may account for the increased photocurrent of Au/GCE in one side. The UV–vis spectroscopy of as-prepared AuNPs was shown in Additional file 1: Figure S2. The absorption peak was located at the wavelength of 597 nm, which is approximate to the wavelength in some previous reports [31, 32]. In detail, the Au nanoparticles could harvest the energy of visible light [33]. Due to the SPR excitation, the accumulation of hot electrons can transiently occupy the higher empty states above the Fermi level, and then a quick transfer of electrons from Au to conductive GCE occurs [34]. The oxidation process of UA takes place on the surface of Au nanoparticles, where the UA molecular transfers two electrons to the Au nanoparticle. In another side, after modified with Au nanoparticles, the electron transfer rate was greatly promoted.

### Effect of Gold Quantity

**Fig. 4:**
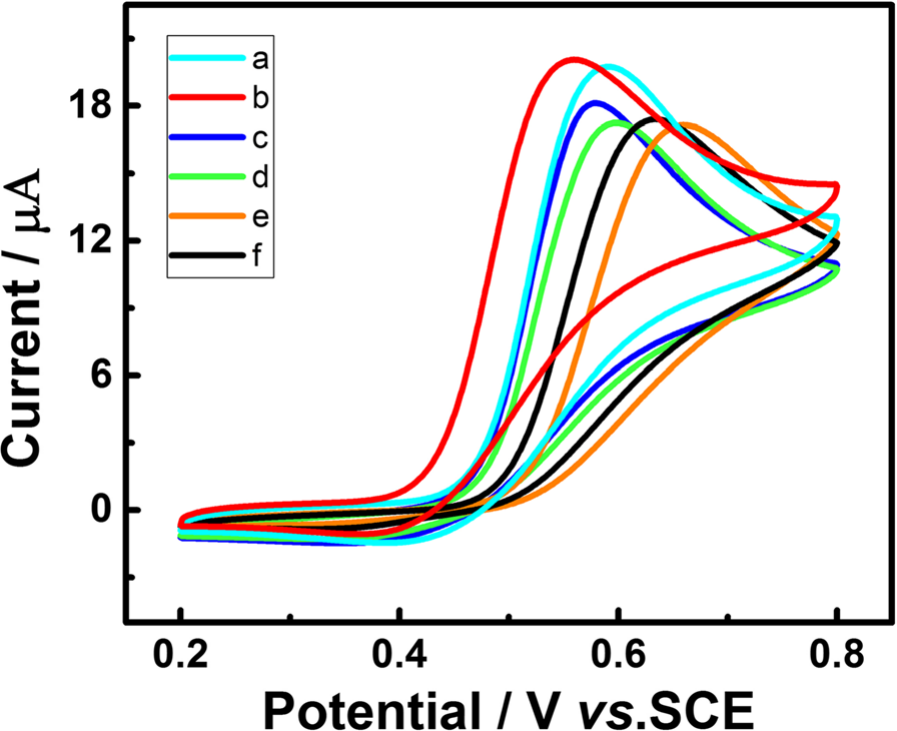
CVs of Au/GCE electrodeposited with different quantity of gold: 57.8 *(a)*, 28.9 *(b)*, 9.6 *(c)*, 4.8 *(d)*, 0.96 μg/cm^2^ *(e)*, and bare GCE *(f)* in 0.1 M PBS (pH 7.0) solution containing 1.0 mM UA under visible light illumination. Scan rate 50 mV s^−1^. Depositing potential −0.2 V

The mass loading of Au deposited on the surface of the electrode was controlled by electric quantity, and its influence on electrochemical performance toward UA was evaluated. As can be seen in Fig. 4, when the quantity of gold is only 0.96 μg/cm^2^ (Fig. 4e), the peak current was almost the same as that of bare GCE (Fig. 4f), even a little decline. However, as the quantity of gold extended from 4.8 to 28.9 μg/cm^2^ (Fig. 4 (d), (c), and (b)), the peak current increased successively and the oxidation peak potential was more negative. Continue to aggrandize the amount of gold to 57.8 μg/cm^2^ (Fig. 4a), the peak current manifested a slight decrease and the peak potential turned to be more positive. It mainly due to the fact that the quality of 28.9 μg/cm^2^ is the best suitable surface area for UA reaction on Au/GCE. In primarily, the bare GCE or low Au nanoparticles covered GCE cannot support the enough activity sites and has insufficient surface area for the reaction. Meanwhile, the GCE, which is covered with excess Au nanoparticles, will slow down the speed of electron transfer from Au/GCE to UA. Therefore, we chose the Au/GCE with gold quantity of 28.9 μg/cm^2^ as the work electrode in the electrochemical detection process.

### Effect of pH Value

**Fig. 5:**
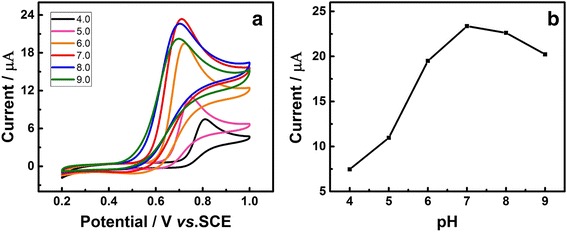
**a** CVs of the Au/GCE in 1 mM UA with different pH PBS solution (pH = 4.0, 5.0, 6.0, 7.0, 8.0, and 9.0), scan rate 50 mV s^−1^. **b** Relationship between the oxidation peak current and pH value

To further evaluate the optimal conditions for UA detection, the influence of pH value on the photo-electrochemical responses of 1 mM UA under visible light illumination was studied. Figure 5a describes the CVs of Au/GCE in 1 mM UA solution, and 0.1 M PBS was used to modulate the pH of each test liquid. Figure 5b shows the relationship between pH value and peak current, from which we can see the maximum value of *I*_pa_ appeared at a pH of 7.0 and subsequently decreased with increasing of the pH from 7.0 to 9.0, which proved that the pH 7.0 is the optimal pH value for UA detection.

### Effect of Scan Rate

**Fig. 6:**
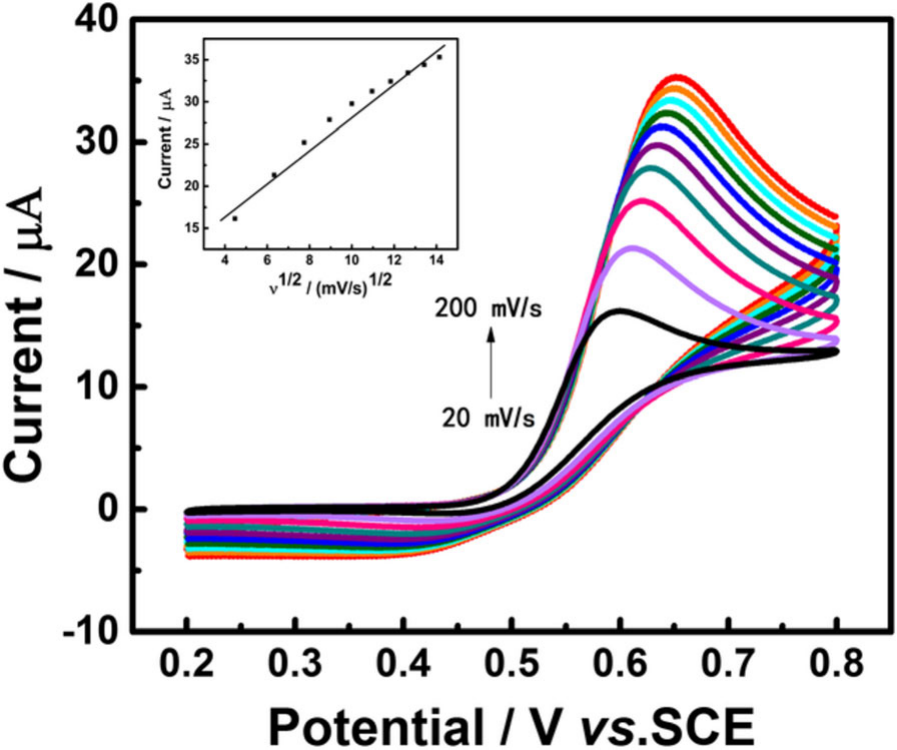
CVs of the Au/GCE in 0.1 M PBS (pH = 7.0) solution containing 1 mM UA at scan rates ranging from 20 to 200 mV s^−1^. *Inset*: calibration curve of peak current vs. v^1/2^

To investigate how scan rate influence the electrochemical response of Au/GCE in UA solution, CVs were operated under different scan rates from 20 to 200 mV s^−1^ with an interval of 20 mV s^−1^. The results were shown in Fig. 6, from which we can see an obvious positive shift in oxidation peak potential as the acceleration of scan rate, indicating that the reaction of UA on the electrode surface is a quasi-reversible chemical process. In other words, the adsorption of UA did not occur on the surface of the electrode in 0.1 M PBS (pH 7.0) solution. Additionally, a linear correlation between anodic peak currents and the square root of scan rates was easily found and the calibration equation is present as *I*_pa_ (μA) = 1.9254ν^1/2^ (mV/s)^1/2^ + 9.3766 (*R* ^2^ = 0.9702), indicating that the reaction rate was determined by the diffusion of UA [35].

### Determination of UA

**Fig. 7:**
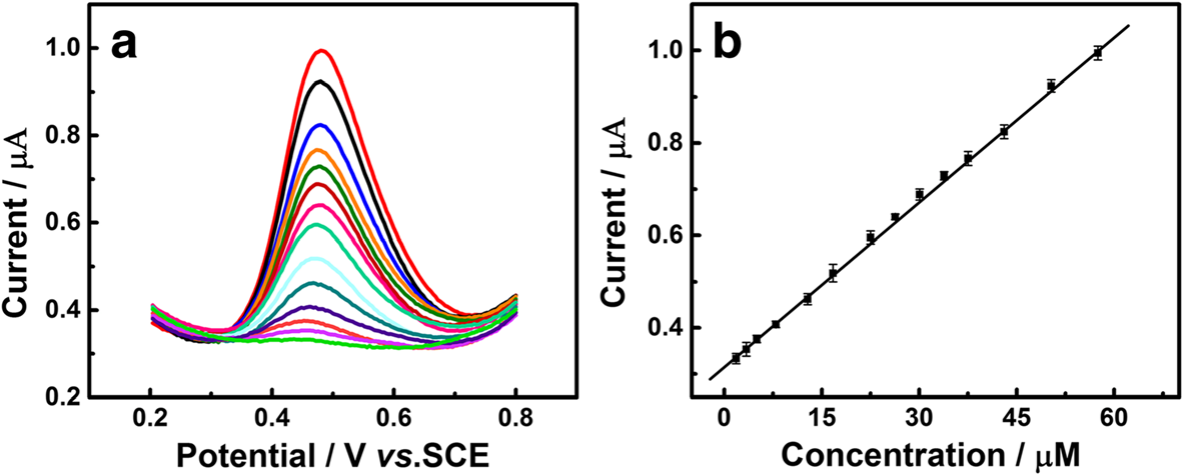
**a** DPV curves of the Au/GCE in 0.1 M PBS (pH 7.0) at different concentrations of UA. **b** Plots of anodic peak currents vs. concentration of UA

Differential pulse voltammetry (DPV), with the features of better resolution and higher sensitivity than CV, has also been employed to the characterization of Au/GCE. There was a proportional relation between oxidation peak current and UA concentration in the range from 2.8 to 57.5 μM (Fig. 7). The linear regression equation can be expressed as *I*_pa_ (μA) = 0.0121*c*_UA_ (μM) + 0.3122 (*R* ^2^ = 0.9987). For comparison, the DPV analysis of Au/GCE toward UA without visible light illumination was shown in Additional file 1: Figure S3, which exhibited a narrower linear range from 3 to 21 μM with the linear regression equation represented as *I*_pa_ (μA) = 0.0112*c*_UA_ (μM) + 0.2766 (*R* ^2^ = 0.9943).All experiments in this section were proceeded under best conditions achieved from previous experimental data. The reusability of Au/GCE was estimated by measuring the CV responses of eight electrodes in 0.1 M PBS (pH 7.0) solution containing 1.0 mM UA under visible light illumination under the same condition. The relative standard deviation of the oxidation peak currents is 7.88%. The stability of Au/GCE under visible light illumination was also checked by performing the modified electrode which was stored a week at 25 °C before the experiment. The peak currents decreased 9.8%. These results above indicate that the modified electrode with visible light illumination shows a well reusability and good stability. And this method could tolerate the general interferences. In the real sample analysis, a recovery of 96.3% was obtained. From the research above, we could make a conclusion that the visible light illuminated Au/GCE shows a potential to be a sensitive electrochemical sensor in the future.

## Conclusions

In summary, homogeneous spherical Au nanoparticles were simply modified on the surface of GCE with constant potential electrodeposition. The SEM images and size distribution histogram revealed that the well-dispersed AuNPs had a narrow size distribution and the average diameter was calculated to be 26.1 nm. This may be conductive to greatly increase the active surface area and ultimately lead to the enhancement of detection efficiency. The electroanalytical results of as prepared Au/GCE under visible light illumination exhibited obvious superiority compared with non-visible light illumination due to the surface plasma resonance (SPR). In addition, this study presents a simple and easy method for detecting the concentration of uric acid, which has the potential to be applied in monitoring other biological substances.

## Additional File

Additional file 1: Figure S1. CVs of Au/GCE and GCE in 2.5 mM Fe(CN)6^3−/4−^+ 0.1 M KCl solution, scan rate 50 mV s^−1^. Figure S2. UV–vis spectroscopy of AuNPs. Figure S3. (a) DPV curves of the Au/GCE in 0.1 M PBS (pH = 7.0) at different concentrations of UA without visible light illumination. (b) Plots of anodic peak currents vs. concentration of UA. (DOCX 342 kb)

## Abbreviations

AuNPs: Gold nanoparticles
CE: Capillary electrophoresis
CV: Cyclic voltammetry
DPV: Differential pulse voltammetry
GCE: Glassy carbon electrode
HPLC: High-performance liquid chromatography
SEM: Scanning electron microscope
SPR: Surface plasmon resonance
UA: Uric acid
