# Supplementary material for: The Enhanced Photo-Electrochemical Detection of Uric Acid on Au Nanoparticles Modified Glassy Carbon Electrode
Source: Nanoscale Res Lett. 2017 Jul 14;12:455. doi: 10.1186/s11671-017-2225-3 (PMC5509567; doi:10.1186/s11671-017-2225-3)
Supplement: Additional file 1: Figure S1. — CVs of Au/GCE and GCE in 2.5 mM Fe(CN)63−/4−+ 0.1 M KCl solution, scan rate 50 mV s−1. Figure S2. UV–vis spectroscopy of AuNPs. Figure S3. (a) DPV curves of the Au/GCE in 0.1 M PBS (pH = 7.0) at different concentrations of UA without visible light illumination. (b) Plots of anodic peak currents vs. concentration of UA. (DOCX 342 kb) [file 11671_2017_2225_MOESM1_ESM.docx]

**Additional files**

**The enhanced photo-electrochemical detection of uric acid on Au nanoparticles modified glassy carbon electrode**





**Figure S1**. CVs of Au/GCE and GCE in 2.5 mM Fe(CN)_6_^3-/4-^+ 0.1 M KCl solution, scan rate: 50 mV s^−1^.





**Figure S2.** UV–vis spectroscopy of AuNPs.

**
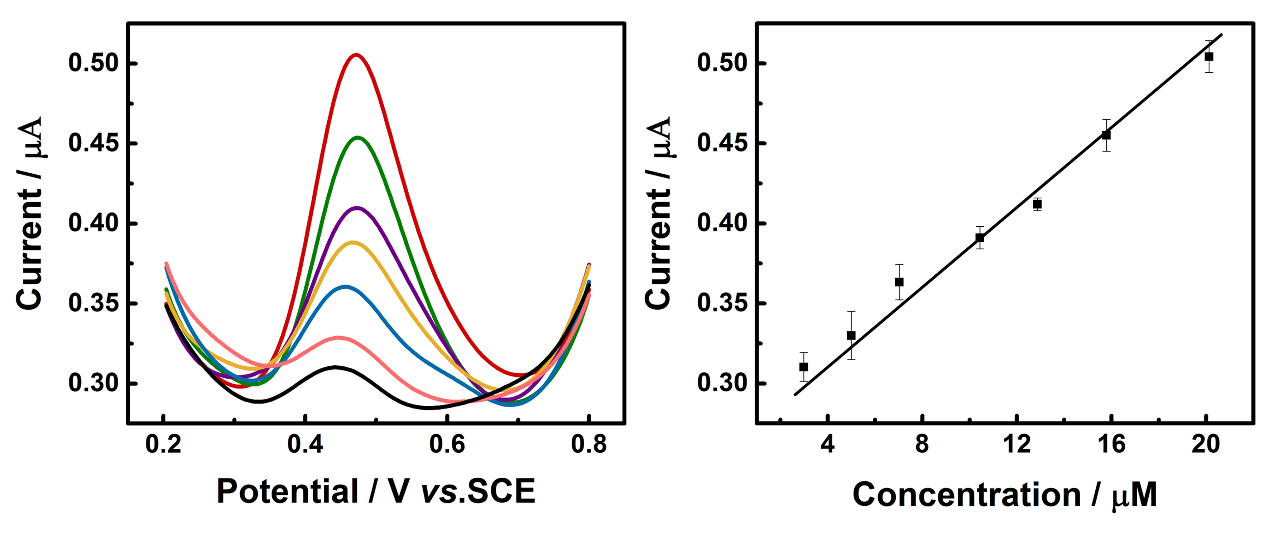
**

**Figure S3.** (a) DPV curves of the Au/GCE in 0.1 M PBS (pH =7.0) at different concentrations of UA without visible light illumination. (b) Plots of anodic peak currents *vs*. concentration of UA.
